# The immediate effect of muscle release intervention on muscle activity and shoulder kinematics in patients with frozen shoulder: a cross-sectional, exploratory study

**DOI:** 10.1186/s12891-017-1867-8

**Published:** 2017-11-28

**Authors:** Yi-Fen Shih, Pei-Wen Liao, Chun-Shou Lee

**Affiliations:** 1 Department of Physical Therapy and Assistive Technology, National Yang-Ming University, 155, Li-Nong Street Sec 2, Pei-Tou District, Taipei, Taiwan 112; 2 Division of Physical therapy, Department of Rehabilitation, Taipei City Hospital-Renai Branch, Taipei, Taiwan

**Keywords:** Frozen shoulder, Muscle activity, Shoulder kinematics, Muscle release

## Abstract

**Background:**

Contractile tissue plays an important role in mobility deficits in frozen shoulder (FS). However, no study has assessed the effect of the muscle release technique on the muscle activation and kinematics in individuals with FS. The purposes of this study were to assess the differences in shoulder muscle activity and kinematics between the FS and asymptomatic groups; and to determine the immediate effects of muscle release intervention in the FS group.

**Methods:**

Twenty patients with FS and 20 asymptomatic controls were recruited. The outcome measures included muscle activity of the upper and lower trapezius (UT and LT), infraspinatus (ISp), pectoralis major (PM), and teres major (TM), shoulder kinematics (humeral elevation, scapular posterior tilt (PT) and upward rotation (UR), shoulder mobility, and pain. Participants in the FS group received one-session of heat and manual muscle release. Measurements were obtained at baseline, and immediately after intervention. Multivariate analysis of variance was used for data analysis. The level of significance was set at α=0.05.

**Results:**

Compared to the controls, the FS group revealed significantly decreased LT (difference =55.89%, P=0.001) and ISp muscle activity (difference =26.32%, P =0.043) during the scaption task, and increased PM activity (difference =6.31%, P =0.014) during the thumb to waist task. The FS group showed decreased humeral elevation, scapular PT, and UR (difference = 35.36°, 10.18°, 6.73° respectively, P <0.05). Muscle release intervention immediately decreased pain (VAS drop 1.7, P <0.001); improved muscle activity during scaption (UT: 12.68% increase, LT: 35.46% increase, P <0.05) and hand to neck (UT: 12.14% increase, LT: 34.04% increase, P <0.05) task; and increased peak humeral elevation and scapular PT during scaption (95.18°±15.83° to 98.24°±15.57°, P=0.034; 11.06°±3.94° to 14.36°±4.65°, P=0.002), and increased scapular PT during the hand to neck (9.47°±3.86° to 12.80°±8.33°, P=0.025) task. No statistical significance was found for other group comparisons or intervention effect.

**Conclusion:**

Patients with FS presented with altered shoulder muscle activity and kinematics, and one-session of heat and manual muscle release showed beneficial effects on shoulder muscle performance, kinematics, mobility, and pain.

**Trial registration:**

Retrospectively registered on Jan 18, 2016 (ACTRN 12616000031460).

**Electronic supplementary material:**

The online version of this article (10.1186/s12891-017-1867-8) contains supplementary material, which is available to authorized users.

## Background

Adhesive capsulitis, also known as frozen shoulder (FS) is a common shoulder disorder with a prevalence of up to 26% of the adult population [1, 2]. It is characterized by chronic shoulder pain, mobility deficits, and functional limitations [1, 2]. Among the dysfunctions, it is mobility deficits that influence a patient’s quality of life the most. Research has shown that mobility deficits of FS affected not only the glenohumeral but also the scapulothoracic articulation, including an abnormal glenohumeral rhythm, excessive scapular anterior tilt and external rotation, and early scapular upward rotation in patients with FS [3–5].

Frozen shoulder has long been considered a condition with pathological features, mainly in non-contractile tissue such as decreased joint capsule capacity, fibrovascular inflammation of joint capsules, and capsule and ligament adhesion [6–8]. Recent publications however suggested that the role of contractile tissue in shoulder mobility deficits and functional limitations might have been overlooked [9, 10]. Mao et al. (1997) found no non-contractile tissue change occurring with improvement of the shoulder range of motion after joint mobilization in patients with FS, and suggested enhanced shoulder mobility might be a result of increased flexibility of the contractile tissues [9]. Hung et al. (2010) found patients with FS had higher muscle stiffness, which was related to the shoulder ROM limitation [10].

Despite the increasing attention on the role of the contractile tissue, only one study investigated the muscle performance of patients with FS [11]. Lin et al. (2005) found patients with FS exhibited imbalanced and hyperactive upper and lower trapezius at various shoulder elevation positions [11], and suggested these altered scapular muscle activations might contribute to compensatory scapular dyskinesis [12–14]. However, the relationship between muscle activity and scapular kinematics during functional movement has never been assessed in patients with FS.

Muscle release (or myofascial trigger point release), defined as deep pressure to areas of local tenderness, has been used to treat chronic painful muscle spasms, decrease pain, and increase range of motion [15, 16]. Researchers have demonstrated that this technique effectively improved mechanical muscle properties in individuals with chronic shoulder pain [15]. However, no study has assessed the effect of the muscle release technique on the muscle activation and kinematics in individuals with FS.

The objectives of this study are to [1] compare differences in shoulder muscle activity and kinematics between patients with FS and matched asymptomatic subjects; and [2] determine the effects of one-session of muscle release techniques on shoulder muscle activity and kinematics during three functional movement tasks. Our hypotheses are [1] patients with FS would show altered shoulder muscle activity and kinematics compared with matched asymptomatic subjects; [2] one-session of muscle release intervention would immediately improve shoulder muscle activity and kinematics, shoulder range of motion, and pain in the patient group.

## Methods

### Participants

This is an exploratory and cross-sectional study. Twenty patients suffering from unilateral FS and 20 asymptomatic subjects were recruited from the Taipei area, Taiwan. Based on data from previous studies [11], the sample size of 20 participants in each group was considered adequate to detect between group and within group difference with a power of 80% (significance level = 0.05). Inclusion criteria for the patient group included [1] medical diagnosis of FS (by physicians specializing in Orthopedics or Physical Medicine), [2] pain and stiffness over the affected shoulder region for more than three months, [3] no resting pain or night pain in the affected shoulder region, and willingness to participate in this study. Asymptomatic subjects were recruited with age, gender, BMI (body mass index), and tested shoulder matched with the FS group. Exclusive criteria for all subjects were: [1] history of surgery or fracture of the shoulder complex, [2] shoulder joint dislocation, [3] rheumatoid arthritis, [4] osteoarthritis of the particular shoulder joint, [5] cervical radiculopathy, or [6] shoulder ROM (range of motion) limitation due to stroke or spinal cord injury.

Ethical clearance was obtained from Taipei City Hospital Institutional Review Board (IRB number TCHIRB-1030708-E). The testing procedures were fully explained, and written informed consent was obtained for all participants before commencement of the study. This experiment was conducted at the Musculoskeletal and Sports Sciences Laboratory, National Yang-Ming University, Taiwan.

### Instrumentation

We used an 8 channel FM/FM Telemetric EMG system (Telemyo 2400, Noraxane USA) to record muscle activation. The input impedance of the system was 10 MΩ, common mode rejection ration of 85 dB and gain of 2000. All signals were converted by an analog-to digital (A/D) converter (NI PCM-CIA 6036 E, USA; 12-bit resolution) and the sampling rate was 1500 Hz. The raw electromyography (EMG) signals were collected by silver/silver chloride pre-gelled surface electrodes (Blue Sensor P-00-S, Ambu Inc., USA) with 2 cm inter-electrode distance. The electrodes were placed at the midway between the spinous process of 7th cervical vertebra, the center of the acromion, and the spinous process of eighth thoracic spine for the lower trapezius (LT); at the center of the infraspinous fossa for the infraspinatus muscle (ISp); at the middle of the muscle belly along the lateral border of the scapula for the teres major (TM) [17], and 3.5 cm medial to the anterior axillary line for the pectoralis major (PM) [18]. The ground electrode was attached to the head of the clavicle.

The Liberty electromagnetic tracking system (Polhemus, Colchester, USA) was used to collect the three-dimensional shoulder kinematics at 120 Hz. Three sensors were used and attached to the spinous process of the 7th cervical vertebra, posterior-lateral acromion, and lower 1/3 aspect of the humerus with adhesive tape [19]. A pen-like stylus was used to digitize the palpated bony landmarks to define the anatomical coordinate systems based on the suggestions of International Society of Biomechanics [19].

### Muscle release intervention

**Fig. 1:**
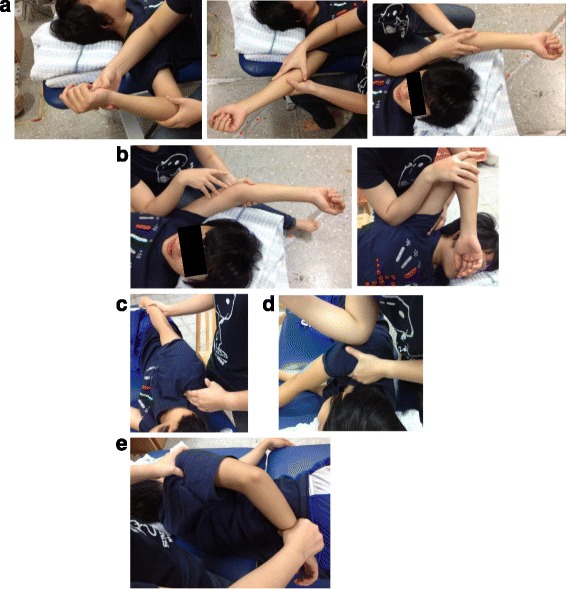
Positions for the muscle release intervention: **a** Pectoralis major: supine with shoulder positioned at external rotation, abduction, and flexion; **b** Teres major: supine with shoulder flexion; sidelying with shoulder abduction; **c** Upper trapezius: sidelying with arm relaxed by the side; **d** Posterior deltoid: sidelying with arm horizontal adduction; **e** Infraspinatus: sidelying with shoulder internal rotation, thumb to the waist

The FS group received the electrical heating pad for 15 min at a temperature between 42 and 45 degrees Celsius, followed by one-session of manual muscle release (PM, UT, ISp, TM, and posterior deltoid) for about 30 min right after the initial examination. The target muscle of intervention was positioned in the lengthened position, while the physical therapist used the elbow and fingers to give sustained pressure directly on the most tender points at the muscle belly for 60 to 90 s until the physical therapist felt the target muscles start to release or the patient felt the pain decrease (Fig. 1) [15, 16]. The same licensed physical therapist (Liao PW), who has had more than two years practicing this technique performed all the treatments.

### Procedures

**Fig. 2:**
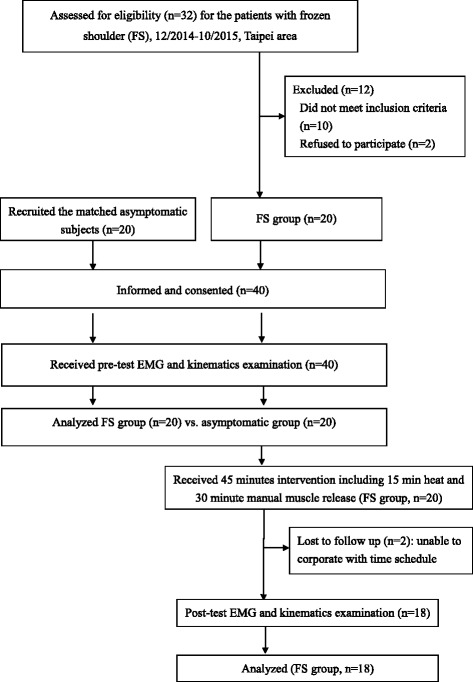
Flow of the study. (EMG: electromyography)

Figure 2 shows the flow of the study. All subjects performed a 10 min warm-up with a hand cycle. Subjects were free to choose their comfortable speed and resistance level. This was followed by measurement of surface EMG (PM, IPs, TM, UT, and LT) and shoulder kinematics (humeral elevation, and scapular tilting and upward rotation) during three functional tasks in the sitting position with the trunk well stabilized by the chair, belt, towel, and foam to avoid any compensatory trunk motion. The functional tasks were: scaption (arm lift in the scapular plane as far as they could), hand to neck (touching the back of the neck using the palm), and thumb to waist (touching the thumb to the back of the waist at the level of the 12th thoracic spine). Before data recording, subjects were asked to practice three to five times to ensure no compensation occurred during the task. Each task was performed at subjects’ comfortable speed three times with 30 s rest in between.

Afterwards, the subject was asked to hold a one-kilogram weight at 80° scaption for five seconds three times while the EMG data of the UT, LT, and ISp during these reference voluntary contractions (RVCs) were recorded [14]. We used a 1 Kg weight for the RVCs because our participants with FS found any weight more than 1 Kg too painful or too heavy to hold, and the RVC normalization method was able to produce reliable EMG data for UT, LT, and ISp without over-fatiguing the participants with shoulder pain [14]. The EMG data during three repetitions of maximum voluntary contractions (MVCs) of the PM and TM were assessed using the standard manual muscle testing methods [17, 20]. After a three minute rest, the measurements of shoulder active and passive ROM (flexion, abduction, external and internal rotation) were performed in the supine position using a universal goniometer with an inclinometer (Table 1) [21]. The data of test-retest repeatability of the goniometer measurement including intraclass correlation coefficient (ICC(3,3)), standard error or measurement (SEM), and minimal detectable change (MDC_95_) are summarized in Additional file 1: Table S1. Three measurements in each direction were performed and averaged for further analysis. A 10 cm visual analog scale (VAS) was used to measure the pain intensity (accuracy = 1 mm) in the FS group, with 0 = no pain and 10 = worse pain.

**Table 1:** Description of the anatomical landmarks for the goniometric measurements including the position of the stable arm, moving arm, and axis

	Axis	Stable arm	Moving arm
Flexion	Lateral aspect of greater tubercle	Axillary line	Lateral epicondyle
Abduction	Anterior aspect of the acromial process	Axillary line	Medial epicondyle
External rotation	Olecranon process	Perpendicular to the ground	Ulnar styloid process
Internal rotation	Olecranon process	Perpendicular to the ground	Ulnar styloid process

After the initial assessment, the locations of the electrodes and motion sensors were marked on the skin, and the electrodes and sensors were removed before intervention. The FS group then received the muscle release intervention, followed by re-attaching the electrodes and motion sensors and the post-treatment assessment of the muscle activity, shoulder kinematics, shoulder ROM, and pain. All assessment and interventions were performed by a licensed physical therapist with more than two years of clinical experience with our assessment and intervention methods.

### Data reduction

The EMG raw data were band-pass filtered (20 to 500 Hz), rectified, and root-mean-squared (RMS) with a window of 50 ms. These data were then normalized by the RVC (UT, LT, ISp) or MVC (PM, TM) EMG data recorded at the baseline, and the mean normalized values were calculated for each task. Data of the three trials were averaged. The measurement of the surface EMG showed good test-retest reliability with ICCs [3] between 0.71 and 0.98 (Additional file 1: Table S1).

The Motion Monitor® (Innovative Sport Training, Inc., Chicago, USA) was used to analyze kinematic data. The Euler angles of the rotational matrices of the humerus and scapula related to the thorax were then calculated. The humeral movement was described firstly about the Y-axis of the thorax (the plane of elevation), the X-axis of the humerus (humeral elevation), and then the Y-axis of the humerus (humeral internal/external rotation). Scapular rotations along the Z-axis defined upward rotation (+), and along the X-axis defined scapular posterior tilt (+) [19]. The peak value of humeral elevation, scapular tilt and upward rotation during functional tasks were obtained and averaged for further comparison. The measurement of shoulder kinematics had a moderate to good test-retest reliability (ICC (3,3) = 0.63~0.99) (Additional file 1: Table S1).

### Data analysis

SPSS 16.0 (SPSS Inc., Chicago, USA) was used for data analysis. The level of significance was set at .05. The multivariate analysis of variance (MANOVA) was used to compare the differences in EMG activity, shoulder kinematics, and shoulder ROM between the FS and the asymptomatic group. The repeated-measure MANOVA was used to compare all the outcome variables before and after the intervention in FS group.

## Results

Twenty patients between the ages of 42 and 65 with unilateral FS (52.85 ± 5.95 years) were recruited, and 20 age, gender, arm dominance matched asymptomatic subjects (53.15 ± 7.14 years) participated in the study. The affected shoulder (twelve dominant shoulders and eight non-dominant shoulders) were tested in the patient group, and the side-matched shoulder were tested in the asymptomatic group. Two patients did not finish the post-test examination because of the time restraints. Descriptive data of the subjects’ characteristics are summarized in Table 2. None of the patients were currently in therapy. Most of them described difficulty in functional movement such as hair combing and hand to the back.

**Table 2:** Baseline characteristics of the subjects with frozen shoulder (FS) (n = 20) and asymptomatic subjects (*n* = 20)

	FS group Mean (SD)	Asymptomatic group Mean (SD)	*P*-value^a^
Age (years)	52.85 (5.95)	53.15 (7.14)	0.89
Gender (male/female)	8 M/12F	8 M/12F	1.00
BMI (kg/m^2^)	22.21 (3.43)	23.32 (3.20)	0.30
Affected or tested shoulder (dominant/ non-dominant)	12/8	12/8	1.00
Duration of symptoms (months)	8.08 (3.09)	–	–

SD: standard deviation

BMI = body mass index (height/weight^2^)

^a^Between group comparisons were assessed using independent t test for continues variables, and chi-square test for the nominal variables. The level of significance was set at *p* < .05

Table 3 summarizes the EMG data before and after the intervention. During scaption, the IPs (81.04 ± 16.75% v.s. 107.36 ± 44.45%, *P* = 0.043) and LT (100.22 ± 29.06% v.s. 156.11 ± 63.61%, *P* = 0.001) were less active in the FS than the asymptomatic group. The FS group exhibited significantly higher PM muscle activation (12.84 ± 9.11% v.s. 6.53 ± 4.75%, *P* = 0.014) during the thumb to waist task as compared to the asymptomatic group. After muscle release intervention, both UT and LT muscle activity improved significantly during scaption (UT: 12.68% increase, *P* = 0.032; LT: 35.46% increase, *P* = 0.022) and hand to neck (UT: 12.14% increase, *P* = 0.041; LT: 34.04% increase, *P* = 0.017) task.

**Table 3:** Comparisons of shoulder muscle activity between the FS (frozen shoulder) group (*n* = 20) and asymptomatic group (n = 20) at baseline; and before and after one-session muscle release intervention in the FS group (*n* = 18)

Muscle activity (%)	Asymptomatic group Mean (SD)	FS group		*P*-value^a^	*P*-value^b^
		Pre-test Mean (SD)	Post-test Mean (SD)		
Scaption task					
Pectoralis major	7.42 (5.87)	6.90 (3.81)	6.42 (3.73)	0.947	0.548
Infraspintus	107.36 (44.45)	81.04 (16.75)	80.05 (18.19)	0.043*	0.911
Teres major	10.54 (4.91)	11.81 (7.86)	10.37 (5.34)	0.968	0.203
Upper trapezius	136.41 (77.08)	103.24 (30.87)	115.92 (43.93)	0.265	0.032*
Lower trapezius	156.11 (63.61)	100.22 (29.06)	135.68 (55.30)	0.001*	0.022*
Hand to neck task					
Pectoralis major	6.41 (4.47)	6.44 (3.36)	6.48 (3.88)	0.640	0.786
Infraspintus	108.24 (38.29)	85.81 (25.51)	87.59 (35.15)	0.091	0.730
Teres major	10.63 (4.69)	10.89 (6.53)	9.61 (4.23)	0.565	0.359
Upper trapezius	85.50 (28.55)	70.05 (17.89)	82.19 (30.60)	0.072	0.041*
Lower trapezius	129.50 (48.75)	102.09 (32.93)	136.13 (53.72)	0.052	0.017*
Thumb to waist task					
Pectoralis major	6.53 (4.75)	12.84 (9.11)	11.48 (8.26)	0.014*	0.355
Infraspintus	62.93 (30.35)	57.07 (26.57)	57.10 (29.71)	0.602	0.455
Teres major	9.75 (5.93)	13.63 (13.00)	9.64 (5.22)	0.289	0.086
Upper trapezius	22.07 (15.96)	22.75 (18.30)	23.72 (17.29)	0.718	0.941
Lower trapezius	25.62 (22.07)	20.75 (18.49)	23.91 (16.94)	0.314	0.254

SD: standard deviation

^a^Multivariate analysis of variance (MANOVA) was used to analyze the differences between the FS and asymptomatic group;

^b^Repeated measures MANOVA was used to analyze the effect of muscle release treatment on muscle activity in the FS group

*The level of significance was set at *p* < .05

Compared to the asymptomatic group, individuals with FS exhibited decreased peak humeral elevation during the scaption (130.54° ± 8.64 v.s. 95.18° ± 15.83, *P* < 0.001) and hand to neck tasks (117.72° ± 8.98 v.s. 95.73° ± 12.09, P < 0.001), and decreased shoulder extension during the thumb to waist task (56.24° ± 7.09 v.s. 48.03° ± 9.79, *P* = 0.004). Peak scapular posterior tilt was significantly impaired in the FS group during the scaption (21.24°+4.51 v.s. 11.06°+3.94, P < 0.001) and hand to neck tasks (16.74°+4.74 v.s. 9.47°+3.86, P < 0.001), while peak scapular upward rotation was only affected during scaption (34.92° ± 9.28 v.s. 28.19° ± 7.74, *P* = 0.017) (Table 4). After muscle release intervention, the FS revealed significant improvement in peak humeral elevation and scapular posterior tilt during the scaption (95.18° ± 15.83° to 98.24° ± 15.57°, *P* = 0.034; 11.06° ± 3.94° to 14.36° ± 4.65°, *P* = 0.002) and scapular posterior tilt during the hand to neck (9.47° ± 3.86° to 12.80° ± 8.33°, *P* = 0.025) tasks (Table 4).

**Table 4:** Comparisons of shoulder kinematics between the FS (frozen shoulder) group (*n* = 20) and asymptomatic group (n = 20) at baseline; and before and after one-session muscle release intervention in the FS group (*n* = 18)

Shoulder kinematics (°)	Asymptomatic group Mean (SD)	FS group		*P*-value^a^	*P*-value^b^
		Pre-test Mean (SD)	Post-test Mean (SD)		
Scaption task					
Humeral elevation	130.54 (8.64)	95.18 (15.83)	98.24 (15.57)	<.001*	0.034*
Scapular PT	21.24 (4.51)	11.06 (3.94)	14.36 (4.65)	<.001*	0.002*
Scapular UR	34.92 (9.28)	28.19 (7.74)	30.17 (7.96)	0.017*	0.209
Hand to neck task					
Humeral elevation	117.72 (8.98)	95.73 (12.09)	98.61 (16.06)	<.001*	0.275
Scapular PT	16.74 (4.74)	9.47 (3.86)	12.80 (8.33)	<.001*	0.025*
Scapular UR	26.45 (6.99)	30.30 (13.43)	26.12 (5.81)	0.262	0.102
Thumb to waist task					
Humeral elevation	−56.24 (7.09)	−48.03 (9.79)	−47.61 (9.49)	0.004*	0.742
Scapular PT	−27.03 (7.06)	−27.49 (7.93)	−26.96 (8.67)	0.846	0.633
Scapular UR	−5.30 (3.60)	−4.86 (4.12)	−4.85 (3.68)	0.719	0.639

SD: standard deviation; PT: posterior tilt; UR: upward rotation

^a^Multivariate analysis of variance (MANOVA) was used to analyze the differences between the FS and asymptomatic group;

^b^Repeated measures MANOVA was used to analyze the effect of muscle release treatment on shoulder kinematics in the FS group

*The level of significance was set at *p* < .05

The between group comparisons showed that patients with FS were less mobile in every direction of shoulder movement (Table 5). One-session of muscle release treatment significantly improved shoulder ROM in all directions (*P* < 0.001) (Table 5), which was accompanied by a significant decrease in pain (VAS score 6.23 ± 1.84 to 4.53 ± 1.91, P < 0.001).

**Table 5:** Comparisons of shoulder active and passive range of motion (AROM and PROM), and pain intensity between the FS (frozen shoulder) group (n = 20) and asymptomatic group (n = 20) at baseline; and before and after one-session muscle release treatment in the FS group (n = 18)

	Asymptomatic group Mean (SD)	FS group		*P*-value^a^	*P*-value^b^
		Pre-test Mean (SD)	Post-test Mean (SD)		
AROM					
Flexion	171.53 (7.36)	129.25 (12.56)	139.48 (15.11)	<.001*	<.001*
Abduction	175.98 (10.34)	91.38 (9.89)	98.43 (9.53)	<.001*	<.001*
External rotation	85.00 (8.15)	30.56 (14.13)	39.68 (13.77)	<.001*	<.001*
Internal rotation	74.07 (12.18)	39.92 (14.18)	45.78 (15.22)	<.001*	<.001*
PROM					
Flexion	175.60 (6.53)	133.86 (12.28)	143.89 (14.82)	<.001*	<.001*
Abduction	180.50 (8.74)	96.86 (8.75)	103.43 (9.27)	<.001*	<.001*
External rotation	89.22 (7.44)	36.61 (13.85)	44.83 (13.67)	<.001*	<.001*
Internal rotation	79.22 (11.28)	45.18(14.38)	51.31 (15.29)	<.001*	<.001*
Pain intensity					
VAS scale	–	6.23 (1.84)	4.53 (1.91)	–	<0.001*

SD: standard deviation; VAS: visual analogue scale

^a^Multivariate analysis of variance (MANOVA) was used to analyze the differences between the FS and asymptomatic group;

^b^Repeated measure MANOVA was used to analyze the effect of muscle release treatment on AROM and PROM, and pain intensity in the FS group

*The level of significance was set at *p* < .05

## Discussion

Frozen shoulder is a chronic shoulder disease accompanied by intense shoulder pain and dysfunction. Despite evidence showing that chronic pain could result in altered muscle performance and consequently contribute to mobility deficits and functional limitations of FS [10, 11, 22–24], limited data was available to describe muscle performance and shoulder kinematics in individuals with FS [11]. This current study is the first attempt to investigate shoulder muscle activation and kinematics during dynamic tasks in patients with FS, and to explore the immediately effect of muscle release intervention on shoulder muscle activation, kinematics, mobility, and pain.

In the past, most researchers observed an over-active upper trapezius muscle in patients with shoulder diseases [11, 12, 25]. Our study however found no statistically significant difference in upper trapezius muscle activation between the asymptomatic and FS group. One explanation was that patients suffering from FS went through constant shoulder pain for months, pain of considerably longer duration and more severity than during shoulder impingement or instability. Chronic pain could arouse strong inhibition signals and lead to the hypo-active upper trapezius [25]. In addition, the EMG signal crosstalk from the levator scapulae might contribute to the differences in upper trapezius EMG findings, as the two muscles have a close anatomical position for surface electrodes. This study also identified significantly decreased lower trapezius muscle activity during scaption in patients with FS as compared to the asymptomatic individuals. Although previous studies observed decreased lower trapezius activity in patients with other shoulder dysfunction [14, 25], our findings were contrary to the data reported by Lin et al. that both upper and lower trapezius muscles were hyper-active when patients with FS held their arms at 60°and 120°scaption position [11]. The possible reason for this inconsistency might be due to the present task being performed dynamically, which could lead to different patterns of muscle activation.

This is the first study attempting to describe muscle performance of the infraspinatus and teres major during functional activities in individuals with FS. Our results demonstrated that the FS group had decreased infraspinatus activation during scaption, but we failed to find any group difference in teres major muscle activation. Although muscle activity of the infraspinatus in patients with FS has not been studied previously, researchers have identified impaired infraspinatus in patients with impingement syndrome [26]. The decreased infraspinatus activity found in this study could be related to the decreased trapezius muscle activity during the scaption task in the FS group. If the lower trapezius is not stabilizing the scapula, then the rotator cuff cannot effectively function [26–29].

Our EMG data also showed that the pectoralis major was hyperactive during the thumb to waist task in the FS group. There are several possible reasons for this result. Firstly, most patients complained of pain and tightness over the anterior shoulder region. Researchers have shown that pain can induce muscle spasm and stimulate the γ motor neuron, thus influencing the muscle activity in the dynamic position [22]. Pain around the shoulder joint could have induced higher muscle tension and activity of the pectoralis major. Secondly, pain stimulates the antagonist muscles in order to prevent more injury [23]. The hyperactive pectoralis major might play an antagonistic role in countering the excessive shoulder extension and internal rotation during this task. No other group difference in muscle activation was observed during the thumb to waist task. Because thumb to waist is the task most challenging for shoulder mobility in the internal rotation direction, the scapular stabilizers (trapezius muscles) and the shoulder external rotators (teres major and infraspinatus) were less likely to show between-group differences during this task.

Our data on shoulder kinematics supported the hypothesis that individuals with FS had impaired humeral elevation, scapular posterior tilt and upward rotation during functional tasks such as scaption and hand to neck task. These kinematic changes reflected in patient’s difficulty in activities such as combing hair. Lin et al. (2005) have reported similar results in patients with shoulder dysfunction [13]. The task, thumb to waist, is a difficult movement for patients with FS, and yet this is the first study examining the kinematics during this movement. Our data showed that the FS group presented with insufficient shoulder extension compared with the asymptomatic group (48.03° v.s. 56.24°), which could be linked to the hyperactive pectoralis major during this task.

Previous studies suggested that muscle release intervention could improve blood circulation, decrease pain, modulate the excitability of the α and γ motoneurons, and improve range of movement [15, 16]. Our study showed muscle release combined with local heat intervention resulted in increased upper and lower trapezius muscle activation during the scaption and hand to neck task, approaching the level found in the asymptomatic group (Table 3). The post-intervention changes in trapezius muscle activity were larger than the SEM and MDC_95_ of the muscle activation measurement of these two muscles (Additional file 1: Table S1), except MDC_95_ of the upper trapezius during the hand to neck task. This indicated that these intervention-related muscle activity changes had potential clinical benefits for individuals with FS. This is the first study showing that muscle release intervention with heat could help in facilitating muscle activation. The decrease in pain intensity after our intervention might also account for the normalization of the muscle performance (Table 4). However, the mechanism for this muscle activation normalization effect require further investigations to clarify.

The immediate improvement of upper and lower trapezius muscle activation following muscle release intervention was accompanied by significantly improved humeral elevation and scapular posterior tilt during the scaption and hand to neck task (Table 3). Similar findings have been revealed in the shoulder impingement syndrome study by applying kinesio taping over the lower trapezius muscle [14]. Our kinematic data provided further support that normalization of the contractile tissue component with local heat and manual muscle release helped to facilitate a better pattern of movement in individuals with FS.

Shoulder ROM limitation and pain are the most disturbing problems in patients with FS. Our results indicated that muscle release intervention immediately enhanced both active and passive ROM and decreased pain (Table 5). There are some possible explanations for the intervention effect. Application of local heat and the manual muscle release treatment might improve the circulation and modulate the local chemical circulation. The mechanical stimulation (heat and pressure) of this intervention might reduce the pain sensation by providing pre-synaptic inhibition at the dorsal horn of the spinal cord and [15, 16]. The improvement in pain might help ease muscle spasms and thus result in the increase in shoulder mobility [15, 16]. However, we were unable to discount the placebo effect as there was no control group involved in this study design. Nevertheless, the increases in both active and passive shoulder mobility were greater than the SEM and MDC_95_ of our goniometer measurement. This suggested that the improvement in shoulder mobility following the heat and manual muscle release warrants a clinical effect which needs further research to confirm. In addition, the decrease in pain from 6.2 to 4.5 while statistically significant may not be clinically meaningful. Whether or not the muscle release intervention has a clinical benefit on pain requires further research to confirm.

### Limitations

Our participants with FS had restricted shoulder ROM, and the degree of limitation varied. Therefore, the target of the functional tasks was adjusted when the subjects in the FS group encountered difficulties during the tasks. We used surface EMG to quantify the level of shoulder muscle activity, and thus crosstalk from neighborhood muscles might occur and influence the results. Our outcome measures did not include the important kinematics for FS assessment, the humeral internal/external rotation, due to inadequate measurement accuracy. We did not standardize the speed of movement to avoid triggering too much pain during the assessment. This uncontrolled factor would nevertheless have an impact on the measurement of kinematics and could not be overlooked. Our data showed that the participants in the patient group were not considered severe for frozen shoulder as they averaged 96.86° ± 8.75°and 36.61° ± 13.85°for passive shoulder abduction and external rotation, which could have potentially skewed the results. We did not objectively control some parameters of the manual muscle release intervention, such as the amount of pressure applied or the total duration of the intervention. The present study only assessed the immediate effect of muscle release intervention with no control or placebo group for comparing the effects of intervention. A future study with a long-term intervention and follow up, and a randomized controlled design is needed to determine the clinical value of the muscle release intervention in patients with FS.

## Conclusion

The results of our study suggested that patients suffering from FS exhibited altered muscle activation and shoulder kinematics during functional activities. One-session of local heat and manual muscle release intervention resulted in an immediate improvement in pain, shoulder mobility, muscle activation of the upper and lower trapezius, and humeral elevation and scapular tilt. These findings implied that pain and contractile tissue had an influence on the abnormalities in shoulder mobility and movement control, which should be taken into consideration when managing patients with frozen shoulder.

## Additional file

Additional file 1: Table S1. Reliability data for all the outcome measures. Test/re-test reliability, standard error of measure, and minimal detectable change for the goniometric measurements; test/re-test reliability for the measurement of muscle activity; and test/re-test reliability for the measurement of shoulder kinematics. (DOC 134 kb)

## Abbreviations

BMI: Body mass index
EMG: Electromyography
FS: Frozen shoulder
ICC: Intraclass correlation coefficient
ISp: Infraspinatus
LT: Lower trapezius
MANOVA: Multivariate analysis of variance
MVC: Maximum voluntary contraction
PM: Pectoralis major
PT: Posterior tilt
RMS: Root mean square
ROM: Range of motion
RVC: Reference voluntary contraction
TM: Teres major
UT: Upper trapezius
UT: Upward rotation
VAS: Visual analogue scale
