# Supplementary material for: The immediate effect of muscle release intervention on muscle activity and shoulder kinematics in patients with frozen shoulder: a cross-sectional, exploratory study
Source: BMC Musculoskelet Disord. 2017 Nov 28;18:499. doi: 10.1186/s12891-017-1867-8 (PMC5706296; doi:10.1186/s12891-017-1867-8)
Supplement: Additional file 1: Table S1. — Reliability data for all the outcome measures. Test/re-test reliability, standard error of measure, and minimal detectable change for the goniometric measurements; test/re-test reliability for the measurement of muscle activity; and test/re-test reliability for the measurement of shoulder kinematics. (DOC 134 kb) [file 12891_2017_1867_MOESM1_ESM.doc]

**Additional file 1: Table S1. Reliability data for all outcome measures.**

| Test/re-test reliability, standard error of measure, and minimal detectable change for the goniometric measurements. | | | | | | |
| --- | --- | --- | --- | --- | --- | --- |
|  | Movement | Active range of motion (degrees) | | ICC_(3,3)_ | SEM | MDC |
|  |  | Test 1  Mean (SD) | Test 2  Mean (SD) |  |  |  |
| Dominate arm | FLEX | 172.3 (2.3) | 172.3 (2.2) | 0.96 | 1.06 | 2.08 |
|  | ABD | 185.0 (3.5) | 185.9 (2.8) | 0.85 | 1.53 | 3.00 |
|  | ER | 95.0 (1.6) | 95.2 (3.0) | 0.92 | 1.23 | 2.41 |
|  | IR | 67.1 (12.1) | 66.0 (11.2) | 0.96 | 2.42 | 4.74 |
| Non-dominate arm | FLEX | 168.3 (6.7) | 167.6 (6.7) | 0.97 | 1.11 | 2.18 |
|  | ABD | 183.6(4.4) | 183.6 (3.5) | 0.85 | 1.26 | 2.47 |
|  | ER | 88.2(6.5) | 87.2 (6.2) | 0.97 | 1.38 | 2.70 |
|  | IR | 68.2 (10.3) | 67.2 (9.7) | 0.95 | 1.88 | 3.68 |
|  | Movement | Passive range of motion (degrees) | | ICC_(3,3)_ | SEM | MDC |
|  |  | Test 1  Mean (SD) | Test 2  Mean (SD) |  |  |  |
| Dominate arm | FLEX | 174.8 (1.9) | 175.0 (1.5) | 0.96 | 0.86 | 1.69 |
|  | ABD | 189.4 (2.6) | 189.8 (2.3) | 0.84 | 1.24 | 2.43 |
|  | ER | 99.2 (1.4) | 99.8 (2.3) | 0.89 | 1.17 | 2.29 |
|  | IR | 72.0 (11.8) | 71.4 (11.6) | 0.95 | 2.80 | 5.49 |
| Non-dominate arm | FLEX | 171.1 (5.6) | 170.4 (5.7) | 0.98 | 0.87 | 1.71 |
|  | ABD | 187.4 (3.9) | 188.4 (2.5) | 0.71 | 1.45 | 2.84 |
|  | ER | 92.9 (6.4) | 93.0 (5.6) | 0.94 | 1.70 | 3.33 |
|  | IR | 72.6 (9.2) | 71.2 (9.0) | 0.97 | 1.39 | 2.72 |
| ICC: intraclass correlation coefficient; SEM: standard error of measurements; MDC: minimal detectable change; FLEX: flexion; ABD: abduction; ER: external rotation; IR: internal rotation | | | | | | |

| Test/re-test reliability for the measurement of muscle activity. | | | | |  |
| --- | --- | --- | --- | --- | --- |
| **Muscle activity (%)** | **Test 1**  **Mean (SD)** | **Test 2**  **Mean (SD)** | **ICC (3,3)** | **SEM** | **MDC** |
| **Scaption task** | | | | | |
| Pectoralis major | 7.70 (4.29) | 5.69 (4.00) | 0.97 | 0.05 | 0.14 |
| Infraspintus | 88.99 (24.22) | 90.20 (16.94) | 0.83 | 1.74 | 4.82 |
| Teres major | 14.96 (9.88) | 8.80 (8.3) | 0.99 | 0.06 | 0.17 |
| Upper trapezius | 119.95 (51.26) | 133.29 (41.83) | 0.82 | 4.24 | 11.75 |
| Lower trapezius | 101.78 (49.64) | 114.24 (51.71) | 0.94 | 1.63 | 4.52 |
| **Hand to neck task** | | | | | |
| Pectoralis major | 5.31 (3.80) | 4.78 (3.94) | 0.97 | 0.06 | 0.17 |
| Infraspintus | 99.6 (29.63) | 95.79 (24.01) | 0.78 | 2.91 | 8.07 |
| Teres major | 9.72 (7.26) | 9.24 (5.38) | 0.91 | 0.29 | 0.80 |
| Upper trapezius | 99.39 (38.53) | 106.84 (26.74) | 0.64 | 5.96 | 16.52 |
| Lower trapezius | 85.42 (27.35) | 93.43 (35.72) | 0.83 | 2.81 | 7.79 |
| **Thumb to waist task** | | | | | |
| Pectoralis major | 15.49 (9.28) | 14.22 (7.87) | 0.88 | 0.52 | 1.44 |
| Infraspintus | 45.18 (24.58) | 45.25 (24.66) | 0.92 | 0.94 | 2.61 |
| Teres major | 10.88 (6.85) | 10.16 (11.05) | 0.77 | 1.04 | 2.88 |
| Upper trapezius | 21.52 (26.91) | 15.56 (21.54) | 0.90 | 1.23 | 3.41 |
| Lower trapezius | 20.48 (8.60) | 22.28 (10.24) | 0.91 | 0.43 | 1.19 |
| SD: standard deviation; ICC: intraclass correlation coefficient; SEM: standard error of measurements; MDC: minimal detectable change | | | | |  |

| Test/re-test reliability for the measurement of shoulder kinematics. | | | | | | | | | |  | | |
| --- | --- | --- | --- | --- | --- | --- | --- | --- | --- | --- | --- | --- |
| **Shoulder kinematics (°)** | **Test 1**  **Mean (SD)** | | **Test 2**  **Mean (SD)** | | **ICC (3,3)** | **SEM** | | | **MDC** | | |  |
| **Scaption task** |  | |  | |  |  | | |  | | |  |
| Humeral elevation | 108.95（23.52） | | 107.86（23.48） | | 0.96 | 4.66 | | | 12.91 | | |  |
| Scapular PT | 16.29（9.02） | | 14.73（7.24） | | 0.97 | 0.13 | | | 0.36 | | |  |
| Scapular UR | 30.73（11.63） | | 27.48（11.45） | | 0.94 | 0.37 | | | 1.03 | | |  |
| **Hand to neck task** | |  | |  |  | |  |  | | |  | |
| Humeral elevation | 103.76（19.21） | | 105.52（18.39） | | 0.98 | 0.16 | | | 0.44 | | |  |
| Scapular PT | 16.75（9.79） | | 14.88（8.65） | | 0.89 | 0.50 | | | 1.39 | | |  |
| Scapular UR | 24.97（11.44） | | 22.80（11.25） | | 0.93 | 0.37 | | | 1.03 | | |  |
| **Thumb to waist task** | |  | |  |  | |  |  | | |  | |
| Humeral elevation | -50.49（7.16） | | -47.50（18.44） | | 0.63 | 2.64 | | | 7.32 | | |  |
| Scapular PT | -24.39（6.53） | | -23.81（6.86） | | 0.83 | 0.55 | | | 1.52 | | |  |
| Scapular UR | -5.74（3.70） | | -6.77（3.39） | | 0.76 | 0.42 | | | 1.16 | | |  |
| SD: standard deviation; ICC: intraclass correlation coefficient; SEM: standard error of measurements; MDC: minimal detectable change; PT: posterior tilt; UR: upward rotation | | | | | | | | | | | |  |
